# Replaced Segment 6 Artery From the Gastroduodenal Artery: A Challenging and Rare Anatomical Variation of Hepatic Artery in Pancreaticoduodenectomy

**DOI:** 10.7759/cureus.44605

**Published:** 2023-09-03

**Authors:** Pothugunta S Krishna, Abhijna soori, Raja Kalayarasan, Pottakkat Biju

**Affiliations:** 1 Department of Surgical Gastroenterology, Jawaharlal Institute of Postgraduate Medical Education & Research, Puducherry, IND

**Keywords:** robotic assisted pancreatic surgery, replaced lha, pancreaticoduodenectomy(pd), gda variation, replaced hepatic artery

## Abstract

Variations in the hepatic artery's anatomy can significantly impact planning and executing pancreatic and hepatobiliary surgeries. Of these, the commonest are variations of right and left hepatic arteries originating from superior mesenteric and left gastric arteries, respectively. The anomalous origin of the right hepatic artery from the gastroduodenal artery (GDA) is among the rarest and most challenging anatomy, especially in patients undergoing pancreatoduodenectomy (PD) since GDA ligation is a mandatory step, which may threaten the liver blood supply. We present a 62-year-old male with suspected distal cholangiocarcinoma and plan a robot-assisted pancreatoduodenectomy. Preoperative computed tomography evaluation revealed an anomalous segment 6 artery arising from the GDA and coursing posterolaterally to the common bile duct in the hepatoduodenal ligament. Also, the patient had a replacement left hepatic artery originating from the left gastric artery. The described vascular anomaly has not been previously reported in patients undergoing PD. Awareness of vascular anomalies is the key to performing oncologically radical surgery without increasing bleeding and ischemic complications in patients undergoing complex procedures like PD.

## Introduction

Michel initially described variations in the hepatic arterial anatomy based on his autopsy series of over 200 dissections [1]. Michel’s classification of hepatic arterial anatomy was later updated by Hiatt et al. in 1994 [2]. Advances in imaging modalities, especially multidetector computed tomography (CT), have improved image quality to assess and interpret these variations [3-5]. However, there are even rare arterial variations of the hepatic artery that were not described in Michel's and Hiatt's publications. One of these rare variations includes the replaced hepatic artery originating from the gastroduodenal artery (GDA) [6]. The accurate preoperative evaluation of the foregut vasculature is of immense clinical importance in performing hepatic and pancreaticobiliary surgeries, especially pancreatectomy and liver transplantation [7,8]. Moreover, these variations have to be meticulously searched for and identified to prevent untoward complications like excessive bleeding [9]. These variations also play a crucial role in achieving successful hepatic artery infusion chemotherapy in managing advanced liver cancers [10]. In this case report, we describe a very rare and complex hepatic arterial pattern in a patient with suspected distal cholangiocarcinoma planned for pancreatoduodenectomy. The replaced left hepatic artery (LHA) originated from the left gastric artery, and the right hepatic artery (RHA) supplied segments 5, 8, and 7 of the right lobe of the liver. The segment 6 artery originated from GDA and traversed posterolaterally to the common bile duct in the hepatoduodenal ligament (HDL). The purpose of this report is to bring awareness to hepato-pancreatic and biliary surgeons regarding the existence of such a rare anomaly and its implications for surgical management [11].

## Case presentation

**Figure 1:**
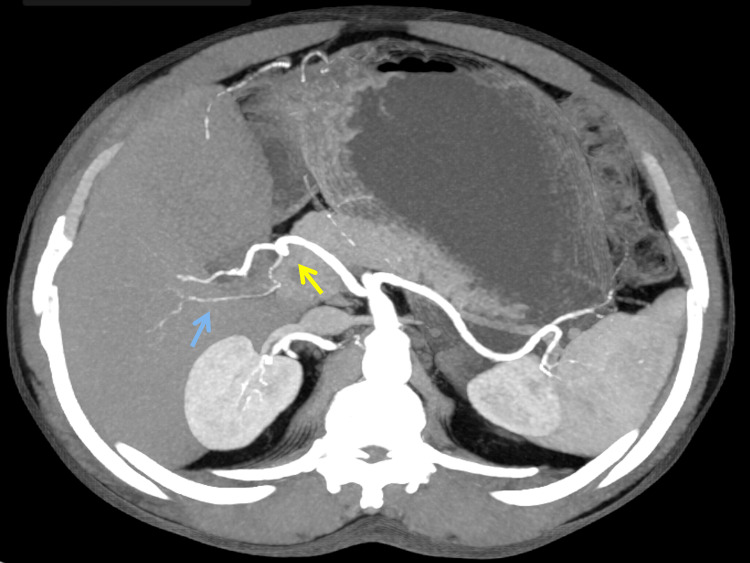
Contrast enhanced CT of the abdomen shows the replaced segment 6 artery (blue arrow) from the gastroduodenal artery (yellow arrow).

**Figure 2:**
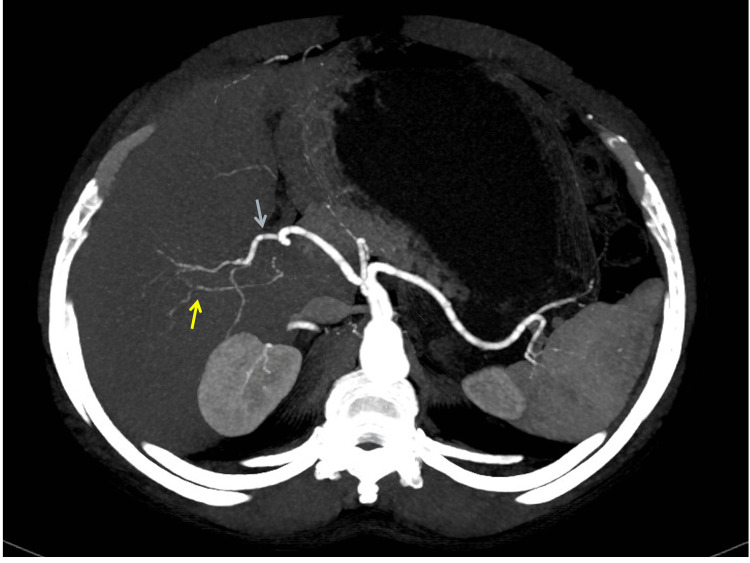
Contrast-enhanced CT of the abdomen shows segments 5, 7, and 8 arteries (yellow arrow) arising from the right hepatic artery (blue arrow).

**Figure 3:**
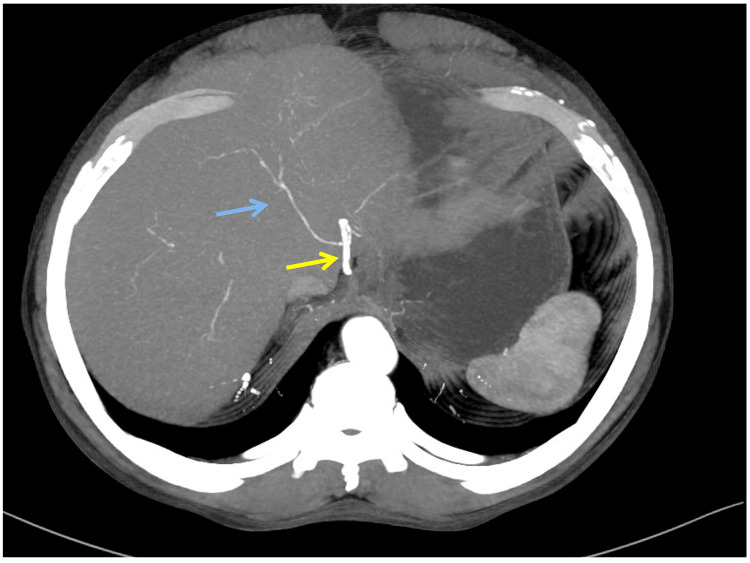
Contrast enhanced CT of the abdomen shows a replacement left hepatic artery (blue arrow) from the left gastric artery (yellow arrow).

A 62-year-old male patient presented with abdominal pain, jaundice, and passage of clay-colored stools for one-month duration. He also had a significant weight loss. On examination, patient had icterus. However, abdominal examination was unremarkable. His laboratory parameters revealed conjugated hyperbilirubinemia with total bilirubin of 9.48 mg/dL and CA19-9 of 75.4 U/mL. Contrast-enhanced CT imaging revealed mild bilobar intrahepatic biliary radicle dilatation. The proximal common bile duct (CBD) was dilated to 13 mm with an abrupt cut-off in the distal CBD. Pancreas was normal in size and attenuation. Arterial anatomy showed a replaced Segment 6 artery originating from GDA (Figure 1), an artery to Segments 5, 7, and 8 of the right lobe of the liver originating from RHA, which is a continuation of the proper hepatic artery (Figure 2), and a replaced LHA originating from the left gastric artery (Figure 3).

**Figure 4:**
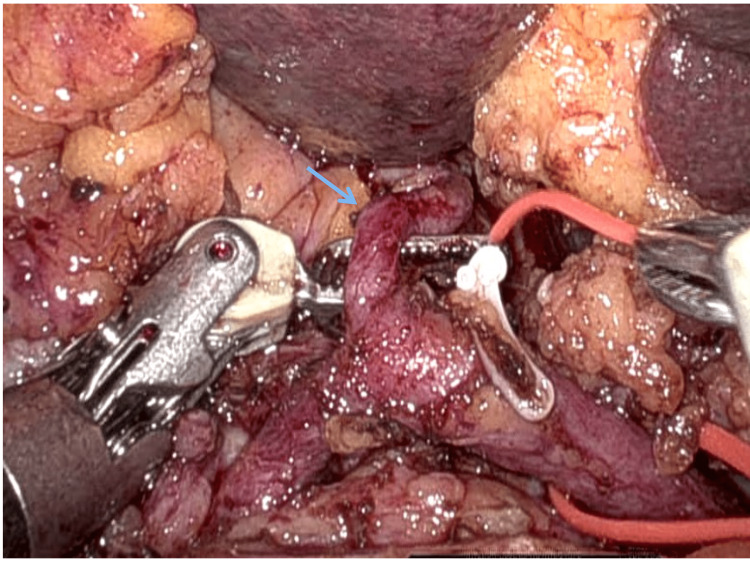
Intraoperative picture depicting a replacement left hepatic artery (blue arrow) from the left hepatic artery.

**Figure 5:**
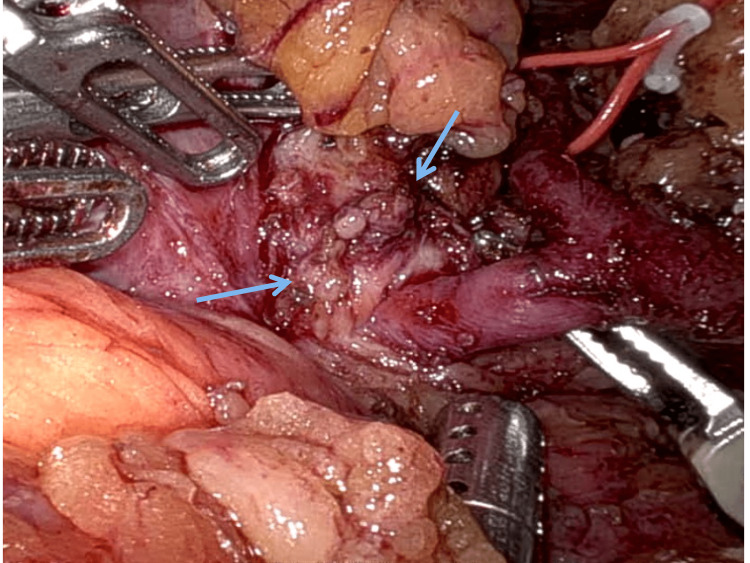
Intraoperative depiction showing intense inflammation in the pancreaticoduodenal groove (blue arrows).

He was suspected to have distal cholangiocarcinoma, and he underwent a robot-assisted pancreaticoduodenectomy. The robot was docked on the right side of the patient. The technique of robotic pancreatoduodenenctomy has been previously reported by the authors [12]. There were marked adhesions between the duodenum and transverse mesocolon, with significant inflammatory changes precluding easy access to the infrapancreatic superior mesenteric vein. The arterial anatomy observed in preoperative imaging was confirmed intraoperatively (Figure 4). In view of dense inflammation in the pancreaticoduodenal grove (Figure 5), safe dissection and isolation of the GDA distal at the origin of segment 6 artery could not be performed.

**Figure 6:**
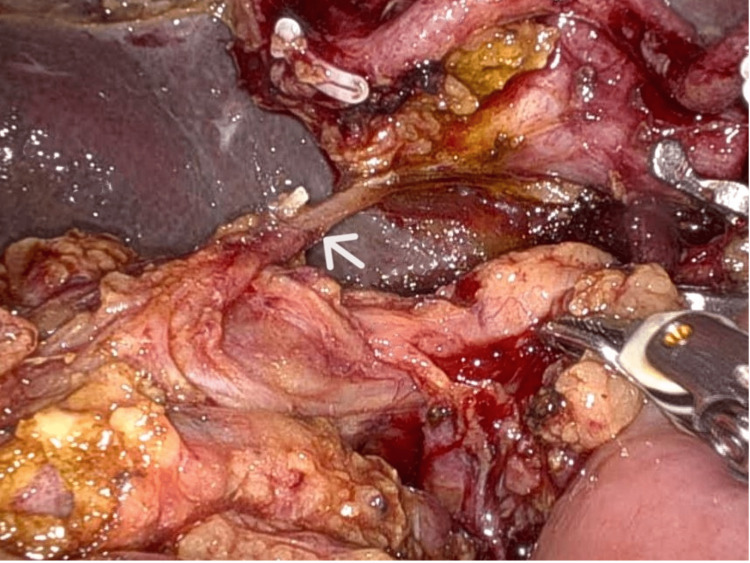
Intraoperative depiction of the replaced segment 6 artery (white arrow) coursing posterolaterally to the common bile duct in the hepatoduodenal ligament.

Hence, GDA was dissected, looped, and divided proximal to the origin of segment 6 artery. The replaced segment 6 artery coursed posterolaterally to the common bile duct in the hepatoduodenal ligament (Figure 6). The segment 6 artery was dissected, looped, and divided close to the hilum to prevent backbleeding from its proximal cut end. The patient had enlarged paraaortic nodes (station 16 b1), which were dissected and sent for biopsy. Jejunum was divided 20 cm distal to duodenjejunal flexure using a laparoscopic linear cutter. After completion of pancreatoduodenectomy, pancreatico-jejunostomy was done by the modified Blumgart's duct-to-mucosa method, and hepatico-jejunostomy was done by the conitnuous technique. Gastrojejunostomy was done using an isolated right jejunal limb. Feeding jejunostomy (FJ) was done 15 cm from the jejunojejunostomy using a 10 Fr infant feeding tube by modified Witzel's technique. The patient had an uneventful postoperative course and was discharged on the eighth postoperative day. The liver function test at discharge was total bilirubin of 1.46 mg/dl and alkaline phosphatase of 134 IU/L. Histopathological evaluation revealed groove pancreatitis.

## Discussion

An anomalous origin of the right hepatic artery is seen in up to 11% to 29% of individuals, and of those variations, the replaced right hepatic artery, taking its origin from SMA, is the most common variation [13]. Michel's classification of hepatic arterial anatomy in 1966 and later the modification by Hiatt in 1994 described the various arterial patterns of the hepatic artery and also reported the incidence of such variations (Table 1). However, these studies didn't describe much rarer varieties of hepatic arterial patterns, which are seen in up to 1.4%-3% of individuals [14]. To overcome such a deficiency, Kobayashi, in 2014, reported a new classification system based on abdominal angiographic images in 1200 cases [15]. In the author's classification system, a replaced right hepatic artery originating from GDA is observed in only 11 cases (0.2%) (Table 2). Preoperative evaluation of such a replaced artery from GDA is of prime importance in patients planned for pancreatoduodenectomy since the ligation of GDA is the critical and mandatory initial step in this complex operation.

**Table 1:** Table showing various hepatic arterial patterns reported by Michel and modified by Hiatt. LHA: Left hepatic artery, LGA: Left gastric artery, RHA: Right hepatic artery, CHA: Common hepatic artery, SMA: Superior mesenteric artery Source [9].

Hepatic arterial pattern	Hiatt’s classification	Michel’s classification
Normal anatomical pattern	Type I	Type I
Replaced LHA from LGA	Type II	Type II
Replaced RHA from SMA	Type III	Type III
Combined type 1 and type 2	Type IV	Type IV
Accessory LHA from LGA	Type II	Type V
Accessory RHA from SMA	Type III	Type VI
Accessory LHA from LGA + accessory RHA from SMA	Type IV	Type VII
Accessory LHA from LGA + replaced RHA from SMA	Type IV	Type VIII
CHA originating from SMA	Type V	Type IX
RHA and LHA originating from LHA	NA	Type X
CHA as direct branch from Aorta	Type VI	NA

**Table 2:** Table showing various hepatic arterial patterns reported by Kobayashi and their incidence. LHA: Left hepatic artery, LGA: Left gastric artery, RHA: Right hepatic artery, CHA: Common hepatic artery, SMA: Superior mesenteric artery Source [15].

Hepatic arterial variation	% of population
Replaced RHA from SMA	5.63%
Replaced LHA from LGA	2.71%
Hepatomesentric trunk	1.04%
Replaced RHA from SMA + replaced LHA from LGA	0.83%
Accessory LHA from LGA	0.62%
Accessory RHA from SMA	0.4%
LHA as direct branch from aorta	0.2%
LHA as direct branch from aorta + Replaced RHA from SMA	0.2%
LHA and RHA originating from SMA	0.2%
LHA originating from CHA and absence of RHA	0.2%
CHA originating from LGA	0.2%
Three hepatic arteries directly originating from aorta	0.2%

The literature describes various strategies to manage such anomalous RHA during complex hepatic and pancreaticobiliary procedures. These techniques include: 1. reconstruction of the replaced RHA 2. Preoperative embolization of the artery 3. Resection in cases where the artery is accessory and involved by the tumor and 4. Neoadjuvant chemotherapy to reduce the size of the tumor and to attempt preservation of the artery [16,17]. Deep knowledge of hepatic arterial anatomy is paramount in performing oncological radical procedures to achieve R0 resection without hindering a safe operation [18].

**Figure 7:**
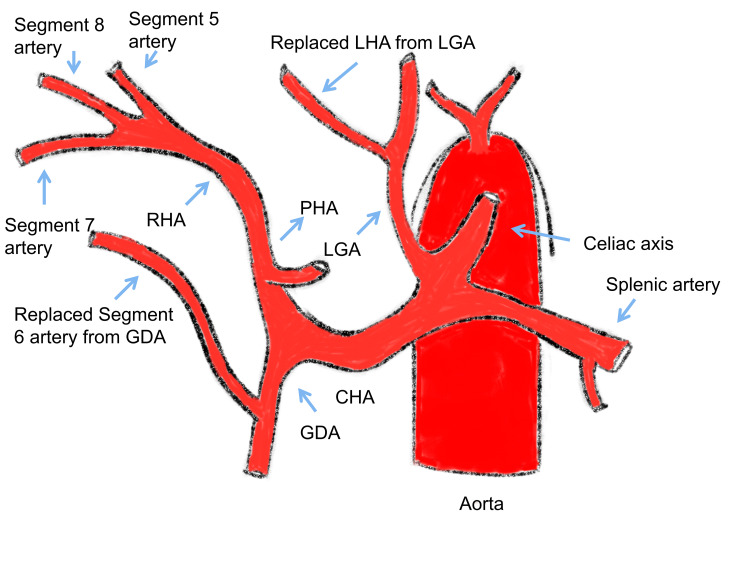
Sketch diagram of the hepatic arterial pattern in the present study. LHA: Left hepatic artery, CHA: Common hepatic artery, LGA: Left gastric artery, PHA: Proper hepatic artery, GDA: Gastroduodenal artery Source [20].

The arterial anatomy described in the present study has not been previously reported (Figure 7). Such hepatic arterial variation could be explained embryologically by the disappearance of the 11th and 12th vitellines and the persistence of the 13th vitelline artery [19]. The importance of knowing this arterial anomaly is to safely dissect the GDA to the origin of segment 6 artery. However, when there is intense inflammation in the pancreaticoduodenal groove, as in the present case, safe dissection of GDA until the origin of segment 6 artery may not be feasible. Also, understanding this anomaly helps to prevent bleeding due to an inadvertent injury to segment 6 of the artery while dissecting the hepatoduodenal ligament. We could safely clip the segment 6 artery without any significant alteration in liver function, as it was only supplying one segment of the right lobe of the liver.

## Conclusions

A rare hepatic arterial anomaly was recognized during computed tomographic angiography in a patient scheduled for pancreaticoduodenectomy for suspected distal cholangiocarcinoma. To our knowledge, the arterial anatomy of the replaced segment 6 artery originating from the GDA and the right hepatic artery supplying segments 5, 7, and 8 of the right lobe of the liver has not been previously reported in the literature. These anomalous hepatic arteries originating from GDA should be kept in mind, especially while performing pancreatoduodenectomy, to avoid ischemic and bleeding complications. In-depth knowledge of the anomalous arterial anatomy has wide application in surgery and interventional radiology.
